# Brain‐Wide Connectivity and Topographic Organization of Leptin Receptor Neurons in the Dorsomedial Hypothalamus

**DOI:** 10.1002/cns.71185

**Published:** 2026-10-01

**Authors:** Yaxin Hao, Xiang Zhang, Danyang Zhao, Haixing Zhong, Yongqiang Chen, Heming Li, Minghui Ren, Mengchu Zhu, Fang Yuan

**Affiliations:** ^1^ Department of Neurobiology Hebei Medical University Shijiazhuang Hebei China; ^2^ Department of Anesthesiology and Perioperative Medicine Xijing Hospital, Fourth Military Medical University Xi'an China; ^3^ Department of Laboratory Diagnostics Hebei Medical University Shijiazhuang Hebei China; ^4^ Hebei Key Laboratory of Brain Science and Brain‐Inspired Intelligence, Hebei Medical University Shijiazhuang Hebei China

**Keywords:** connectivity bias, dorsal part of the DMH, dorsomedial hypothalamus, leptin receptor, neural circuitry

## Abstract

**Objective:**

The dorsomedial hypothalamus (DMH) integrates metabolism and autonomic homeostasis, but whether subnuclear functional topography exists remains unclear. This study compared whole‐brain input–output connectomes of leptin receptor (LepRb) neurons in dorsal DMH (DMD) versus DMH to test for connection‐biased segregation.

**Methods:**

Rabies monosynaptic retrograde tracing and Cre‐dependent AAV anterograde tracing in LepRb‐Cre mice mapped and quantified input–output projections of DMD^LepRb^ and DMH^LepRb^ neurons.

**Results:**

Both DMD^LepRb^ and DMH^LepRb^ neurons received inputs from 58 upstream nuclei and projected to 43 downstream targets across the telencephalon, diencephalon, midbrain, pons, and medulla, forming extensive overlapping networks with similar global architectures. Nevertheless, they showed significant connection weight biases: DMD^LepRb^ preferentially integrated arousal, defensive, and emotional signals and projected to stress/defense‐related nuclei, whereas DMH^LepRb^ preferentially integrated viscerosensory and reward‐aversion signals and projected to metabolic/circadian nuclei.

**Conclusion:**

DMD^LepRb^ and DMH^LepRb^ neurons share highly homologous whole‐brain input–output architecture but exhibit local connection‐weight biases, revealing conserved architecture with local specialization and providing a circuit basis for functional heterogeneity.

## Graphical abstract

Whole‐brain input–output mapping reveals that DMD^LepRb^ and DMH^LepRb^ neurons share a highly conserved global connectivity architecture yet exhibit local connection biases: DMD^LepRb^ neurons preferentially integrate arousal and defensive signals, whereas DMH^LepRb^ neurons favor viscerosensory and metabolic inputs, uncovering a principle of conserved global topology with local functional specialization within the DMH.

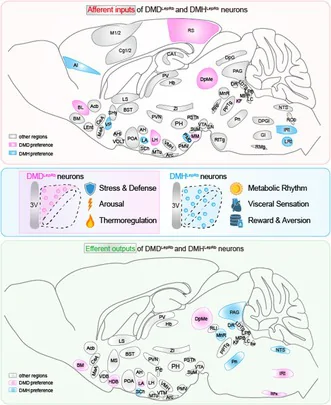

## 1 Introduction

The hypothalamus maintains metabolic and autonomic homeostasis. Among its subregions, the dorsomedial hypothalamus (DMH) links metabolic status with behavioral and autonomic outputs and is associated with obesity [1], stress [2], hypertension [3], and sleep disorders [4]. The DMH is functionally heterogeneous, with distinct subregions and neuronal subtypes [5], regulating energy expenditure [6], feeding [7], thermogenesis [8], autonomic activity [9], and arousal [10]. Its dorsal part (DMD) is more associated with defensive responses, stress‐induced sympathetic activation, and thermogenesis, whereas the ventral part (DMV) is more neuroendocrine [11, 12], suggesting a subnuclear functional topography within the DMH, though the circuit basis remains unclear.

Leptin receptor (LepRb)‐expressing neurons represent a major functional population within the DMH. DMH^LepRb^ neurons regulate feeding, energy metabolism, thermoregulation [13, 14, 15], respiration [16], autonomic activity [17], and sleep–wake regulation [18]. They integrate metabolic and environmental information from the arcuate nucleus (Arc) and preoptic area (POA), and project to autonomic/behavioral centers [19, 20]. Distinct DMH subtypes regulate thermogenesis [21], respiration [16], arousal [22], and stress responses [23] via specific projections. However, most studies treat DMH^LepRb^ neurons as homogeneous, overlooking subregional differences. Functional studies show DMV^LepRb^ neurons rapidly inhibit feeding via GABAergic projections [24], while DMD^LepRb^ neurons specialize in thermogenesis and energy expenditure [15, 25]. Whether DMD^LepRb^ neurons have distinct input–output patterns remains unknown.

Neuronal function depends on connectivity: afferent inputs define the information available to neurons, while efferent outputs determine the downstream systems they regulate. Comparing whole‐brain input–output connectivity of DMD^LepRb^ and DMH^LepRb^ neurons is critical for understanding whether connection‐biased topography exists within the DMH. If both populations share a common global framework but exhibit local biases, it would suggest that the DMH achieves refined regulation through conserved architecture with local specialization. This principle is validated in other nuclei, such as the ventromedial hypothalamus (VMH) [26], and limbic system regions [27] as a key mechanism for generating diverse behavioral outputs. The DMD is a well‐characterized DMH subdivision with documented roles in defense, stress‐evoked sympathetic activation, and thermoregulation—functions distinct from the metabolic and neuroendocrine roles of the DMV. This functional divergence makes the DMD a suitable model to test whether subregional specialization arises from segregated circuits or weighted biases within a shared network, an established strategy in hypothalamic circuit research. This study thus focuses on how DMD^LepRb^ neurons integrate into the broader DMH connectome, rather than comprehensively comparing all DMH subnuclei.

Here, we used Cre‐dependent viral tracing, monosynaptic rabies tracing, anterograde tracing, RNAscope, and whole‐brain quantitative analysis to map and compare the input–output networks of DMD^LepRb^ and DMH^LepRb^ neurons. Our results show that both populations share similar global architectures centered on the diencephalon as a bidirectional hub, yet exhibit significant biases in connection weights: DMD^LepRb^ neurons connect more to autonomic/behavior‐related networks, whereas DMH^LepRb^ neurons connect to metabolic networks. These findings suggest a subnuclear topographic organization within the DMH, where distinct subregions regulate different physiological systems via differential connectivity. Collectively, this study reveals the whole‐brain connectivity principles of DMH^LepRb^ neurons and provides a circuit‐based foundation for understanding DMH functional heterogeneity in metabolism, autonomic functions, and behavior.

## 2 Method

### 2.1 Animals

Adult LepRb‐Cre (Jackson Laboratory, #008320) and C57BL/6J mice (Beijing Vital River), both sexes, 8–10 weeks old, were housed on a 12‐h light/dark cycle (24°C–25°C) with food/water ad libitum.

### 2.2 Viruses and Surgeries

AAV‐EF1α‐DIO‐TVA‐GFP (1.7 × 10^13^ genome copies/mL), AAV‐EF1α‐DIO‐RVG (6.8 × 10^13^ genome copies/mL), RV‐EnvA‐ΔG‐dsRed (5.0 × 10^13^ fluorescence‐forming units/mL) (Brain Case, Wuhan), and AAV‐EF1α‐DIO‐EYFP (1.17 × 10^13^ genome copies/mL, Genechem #19870) were used. Mice under isoflurane were unilaterally injected via glass pipette (25 μm) into DMD (AP −1.94 mm, ML ±0.3 mm, DV −4.9 mm) or DMH (AP −1.94 mm, ML ±0.3 mm, DV −5.25 mm). For retrograde tracing, AAV mixture (1:2, 100 nL, 10 nL/min) was injected; 2 weeks later, 100 nL RV‐EnvA‐ΔG‐dsRed was injected, perfused after 1 week. For anterograde tracing, 100 nL AAV‐EF1α‐DIO‐EYFP was injected, perfused after 4 weeks.

### 2.3 Histology and Immunostaining

Anesthetized mice (urethane 1.8 g/kg i.p.) were perfused with saline then 4% PFA. Brains were post‐fixed (24 h, 4°C), cryoprotected in 30% sucrose (24–36 h), and sectioned at 25 μm (CM1950, Leica). Sections were blocked (5% BSA, 0.25% Triton X‐100 in PBS, 30 min, RT), incubated with chicken anti‐GFP (1:1000, Abcam ab13970) overnight at 4°C, then with goat anti‐chicken Alexa Fluor 488 (1:500, Abcam ab150169) for 2 h at RT, and mounted with Vectashield.

### 2.4 RNAscope Fluorescent In Situ Hybridization

Animals were perfused and brains processed as above. Dehydrated tissues were embedded in O.C.T., snap‐frozen, and cryosectioned. RNAscope Multiplex Fluorescent v2 was performed on 16‐μm sections with antibody co‐detection. After H_2_O_2_ pretreatment and PBS washes, sections were incubated overnight at 4°C with primary antibody in Co‐Detection Antibody Diluent, then treated with Protease Plus. Probes for *Slc17a6*‐C2 (#319171‐C2) and *Slc32a1*‐C3 (#319191‐C3) were hybridized, followed by amplification. Sections were then incubated with goat polyclonal secondary antibody in Co‐Detection Diluent for 30 min at RT, washed, and mounted with Fluoromount‐G (SouthernBiotech).

### 2.5 Single‐Cell RNA Sequencing

Fifteen 8‐week‐old male C57BL/6J mice were anesthetized with urethane (1.8 g/kg, i.p.) and euthanized. Only males were used to minimize variability. Coronal brain slices (200 μm) containing the DMH were prepared using a vibrating microtome (VT1200S, Leica Biosystems, Germany). The DMH was bilaterally microdissected, and pooled tissues were stored at −80°C. Samples were processed using the 10X Genomics Chromium Single Cell 3′ v3.1 platform (OmicsMaster BIOTECHNOLOGY, Guangzhou, China), including: (1) single‐cell suspension preparation; (2) gel beads in emulsion generation and reverse transcription; (3) cDNA amplification and purification; (4) library construction; (5) sequencing on a NovaSeq X Plus platform (PE150).

Subsequent analyses were performed using Seurat (v.5.0.2) in R (v.4.2). Quality control excluded cells with < 500 or > 17,000 unique molecular identifiers (UMIs), > 10% mitochondrial reads, or doublets, retaining 5288 cells (median genes: 2533; median UMIs: 4486.5; median mito%: 0.32%). After normalization (LogNormalize, factor 10,000), the top 2000 variable genes were identified (vst) and scaled while regressing out UMI counts, mito%, and cell‐cycle, followed by principal component analysis (PCA). Using the top 30 principal components, Louvain clustering (resolution 0.1–1.0; resolution 0.5 selected) and UMAP were applied. Markers were defined by Wilcoxon test (log_2_FC > 0.25, adjusted *p* < 0.05).

### 2.6 Cluster Analysis

To examine projection pattern similarity, we performed hierarchical cluster analysis on input/output intensity matrices. Projection intensities were *Z*‐score normalized. A similarity matrix was constructed using Euclidean distances (smaller distance = higher similarity) and visualized as a heatmap. Hierarchical clustering used the complete linkage method, with results displayed as a dendrogram.

### 2.7 Imaging and Data Analysis

Images were captured using a confocal microscope (LSM 800, Carl Zeiss, Germany) and processed with ZEN software (Zeiss, Germany). Neural somata and axonal varicosities were quantified semi‐automatically using ImageJ (U.S. National Institutes of Health, Bethesda, MD, USA). Input ratio = afferent cells in a nucleus/total dsRed^+^ cells (including bilateral afferents). Axonal projection proportion = regional varicosity count/whole‐brain varicosities (reflects relative anatomical varicosity, not functional). Afferent and efferent preference ratios were calculated as the proportion of each input or output to DMD divided by that to DMH. Values > 1, < 1, and = 1 indicate bias toward DMD, DMH, or balanced distribution, respectively. This ratio is a descriptive measure to rank regional biases and is not a formal statistical comparison. Axons with transverse diameters exceeding 0.5 μm were classified as varicose. Data were analyzed using Prism 9.0 (GraphPad Software, San Diego, CA, USA) and presented as mean ± standard error of the mean (SEM). Whole‐brain DMD versus DMH comparisons were assessed by multiple unpaired *t*‐tests, with Benjamini–Krieger–Yekutieli false discovery rate (FDR) correction for multiple comparisons (*Q* = 5%). Differences with *q* < 0.05 were considered significant.

## 3 Results

### 3.1 Anatomical Distribution and Neurochemical Phenotype of LepRb Neurons in the DMD or DMH

To investigate LepRb neuron distribution, we injected AAV‐EF1α‐DIO‐EYFP into the DMD or DMH of LepRb‐Cre mice (Figure 1A), enabling specific labeling (Figure 1B). EYFP‐labeled neurons distributed widely along the DMH rostrocaudal axis, with highest density in the intermediate part; DMD^LepRb^ neurons similarly concentrated in the intermediate region (Figure 1C). Although DMD is considered part of the DMH, its LepRb neurons displayed a more clustered and sharply demarcated pattern, suggesting DMD as a structurally distinct subregion.

**FIGURE 1:**
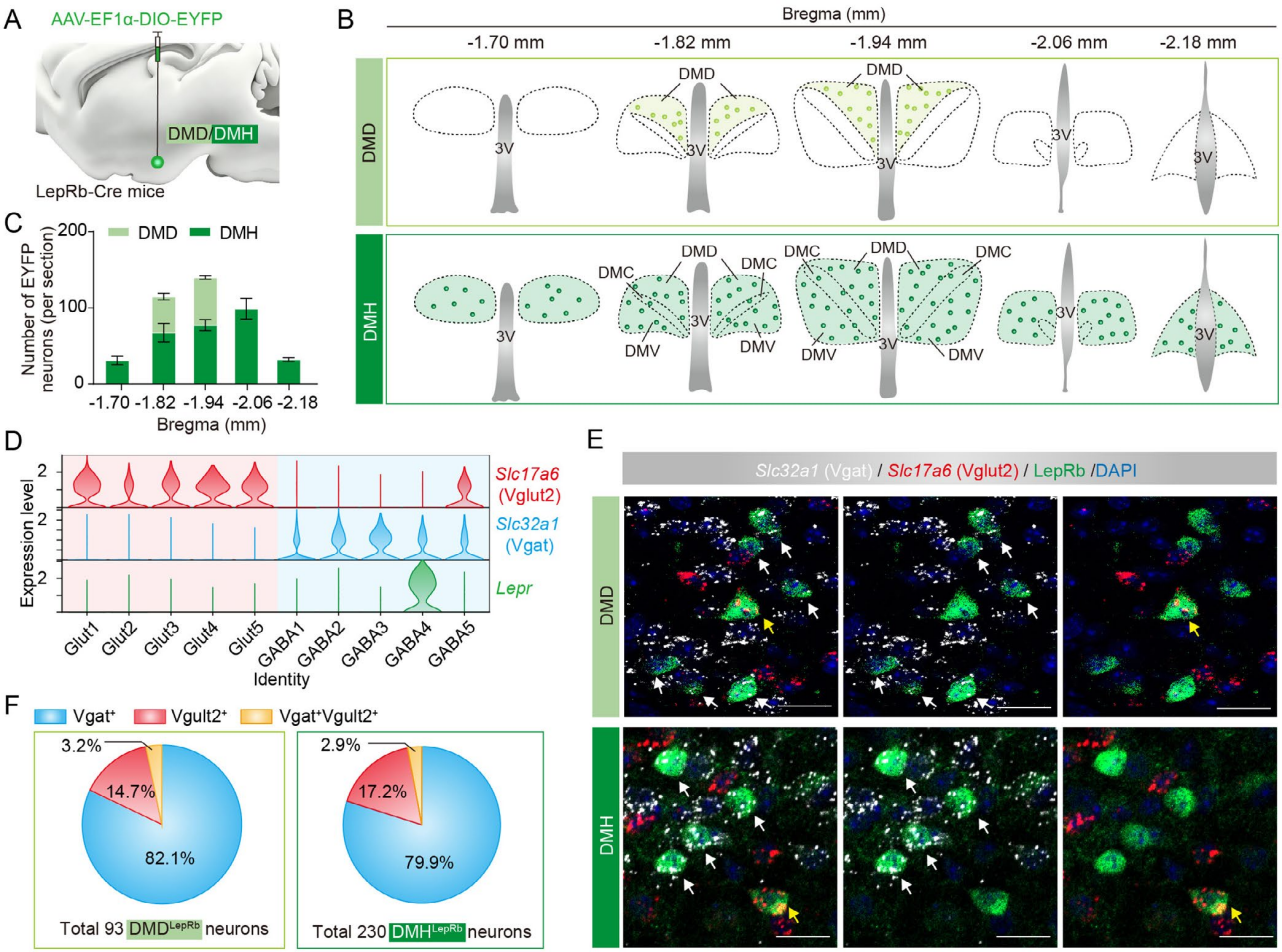
Distribution and neurochemical identity of DMD^LepRb^ and DMH^LepRb^ neurons. (A) Schematic diagram of the viral labeling strategy. (B) Schematic illustrating the rostrocaudal distribution of LepRb‐expressing neurons in the DMD and DMH. Neurons were mapped in coronal sections following immunohistochemical detection. (C) Quantitative rostrocaudal distribution of EYFP^+^ neurons in the DMD and DMH. (D) Violin plots showing the expression of *Slc17a6* (Vglut2), *Slc32a1* (Vgat) and *Lepr* across DMH neuronal clusters. (E) In situ detection of *Slc32a1* and *Slc17a6* in the DMD^LepRb^ and DMH^LepRb^ neurons. White arrows indicate Vgat^+^ LepRb^+^ neurons. Yellow arrows indicate Vglut2^+^ LepRb^+^ neurons. Scale bars, 20 μm. (F) Proportion of Vgat^+^ and Vglut2^+^ in all DMD^LepRb^ and DMH^LepRb^ neurons (*n* = 3). Data represent mean ± SEM.

**TABLE 1:** List of brain region abbreviations.

Abbreviation	Nucleus name	Brain region
Acb	Accumbens nucleus	Telencephalon
AH	Anterior hypothalamic area	Diencephalon
AHi	Amygdalohippocampal area	Telencephalon
AI	Agranular insular cortex	Telencephalon
Arc	Arcuate hypothalamic nucleus	Diencephalon
Bar	Barrington's nucleus	Pons
BL	Basolateral amygdaloid nucleus	Telencephalon
BM	Basomedial amygdaloid nucleus	Telencephalon
BST	Bed nucleus of the stria terminalis	Telencephalon
CA1	Field CA1 of hippocampus	Telencephalon
CeA	Central amygdaloid nucleus	Telencephalon
Cg1/2	Cingulate cortex, area 1/2	Telencephalon
DpG	Deep gray layer of the superior colliculus	Midbrain
DPGi	Dorsal paragigantocellular nucleus	Medulla
DpMe	Deep mesencephalic nucleus	Midbrain
DR	Dorsal raphe nucleus	Midbrain
Gi	Gigantocellular reticular nucleus	Medulla
Hb	Habenular nucleus	Diencephalon
HDB	Nucleus of the horizontal limb of the diagonal band	Telencephalon
IRt	Intermediate reticular nucleus	Medulla
KF	Kolliker‐Fuse nucleus	Pons
LA	Lateroanterior hypothalamic nucleus	Diencephalon
LC	Locus coeruleus	Pons
LDTg	Laterodorsal tegmental nucleus	Pons
LEnt	Lateral entorhinal cortex	Telencephalon
LH	Lateral hypothalamic area	Diencephalon
LPB	Lateral parabrachial nucleus	Pons
LRt	Lateral reticular nucleus	Medulla
LS	Lateral septal nucleus	Telencephalon
M1/2	Primary/secondary motor cortex	Telencephalon
MeA	Medial amygdaloid nucleus	Telencephalon
MM	Medial mammillary nucleus, medial part	Diencephalon
MnR	Median raphe nucleus	Midbrain
MPB	Medial parabrachial nucleus	Pons
MS	Medial septal nucleus	Telencephalon
MTu	Medial tuberal nucleus	Diencephalon
NTS	Nucleus of the solitary tract	Medulla
PAG	Periaqueductal gray	Midbrain
Pe	Periventricular hypothalamic nucleus	Diencephalon
PH	Posterior hypothalamic area	Diencephalon
PMD	Premammillary nucleus, dorsal part	Diencephalon
PMV	Premammillary nucleus, ventral part	Diencephalon
Pn	Pontine reticular nucleus	Pons
POA	Preoptic area	Diencephalon
PPTg	Pedunculopontine tegmental nucleus	Pons
PSTh	Parasubthalamic nucleus	Diencephalon
PV	Paraventricular thalamic nucleus	Diencephalon
PVN	Paraventricular hypothalamic nucleus	Diencephalon
RLi	Rostral linear nucleus of the raphe	Midbrain
RMg	Raphe magnus nucleus	Pons
ROb	Raphe obscurus nucleus	Medulla
RPa	Raphe pallidus nucleus	Medulla
RRF	Retrorubral field	Midbrain
RS	Retrosplenial cortex	Telencephalon
RtTg	Reticulotegmental nucleus of the pons	Pons
SCh	Suprachiasmatic nucleus	Diencephalon
SHy	Septohypothalamic nucleus	Diencephalon
SN	Substantia nigra	Midbrain
SuM	Supramammillary nucleus	Diencephalon
VDB	Nucleus of the vertical limb of the diagonal band	Telencephalon
VMH	Ventromedial hypothalamic nucleus	Diencephalon
VOLT	Vascular organ of the lamina terminalis	Diencephalon
VP	Ventral pallidum	Telencephalon
VTA	Ventral tegmental area	Midbrain
VTM	Ventral tuberomammillary nucleus	Diencephalon
ZI	Zona incerta	Diencephalon

To characterize neurochemical composition, single‐cell RNA sequencing was performed on DMH tissue. Quality control metrics of the sequencing dataset were evaluated (Figure S1A). Based on neurotransmitter markers, neurons were classified into inhibitory (Vgat^+^/*Slc32a1* ^+^) and excitatory (Vglut2^+^/*Slc17a6* ^+^) populations (Figure S1B). Most DMH^LepRb^ neurons expressed *Slc32a1* (Figure 1D and Figure S1C), indicating a predominantly inhibitory phenotype. RNAscope showed that in DMD^LepRb^ neurons, 82.1% co‐expressed *Slc32a1*, 14.7% co‐expressed *Slc17a6*, and 3.2% co‐expressed both. In DMH^LepRb^ neurons, proportions were 79.9%, 17.2%, and 2.9%, respectively (Figure 1E,F). Thus, LepRb neurons in both regions are predominantly GABAergic with similar proportions, suggesting functional differences likely arise from distinct connectivity, not neuronal phenotype.

### 3.2 Rabies Virus Tracing Reveals Monosynaptic Inputs to DMD^LepRb^ and DMH^LepRb^ Neurons

To establish afferent connectivity, we performed cell‐type‐specific monosynaptic rabies tracing in LepRb‐Cre mice. AAV‐EF1α‐DIO‐TVA‐GFP and AAV‐EF1α‐DIO‐RVG were unilaterally injected into the DMD or DMH, followed two weeks later by EnvA‐pseudotyped glycoprotein‐deleted rabies virus (RV‐EnvA‐ΔG‐dsRed) at the same sites (Figure 2A). This distinguished GFP^+^ LepRb neurons (green), dsRed^+^ input neurons (red), and double‐labeled starter neurons (Figure 2B). Both GFP and dsRed signals were detected unilaterally (Figure 2C–E), with no expression in wild‐type mice (Figure 2C, right), confirming Cre‐dependent specificity. These methods enabled visualization and dissection of whole‐brain monosynaptic upstream inputs to DMD^LepRb^ and DMH^LepRb^ neurons.

**FIGURE 2:**
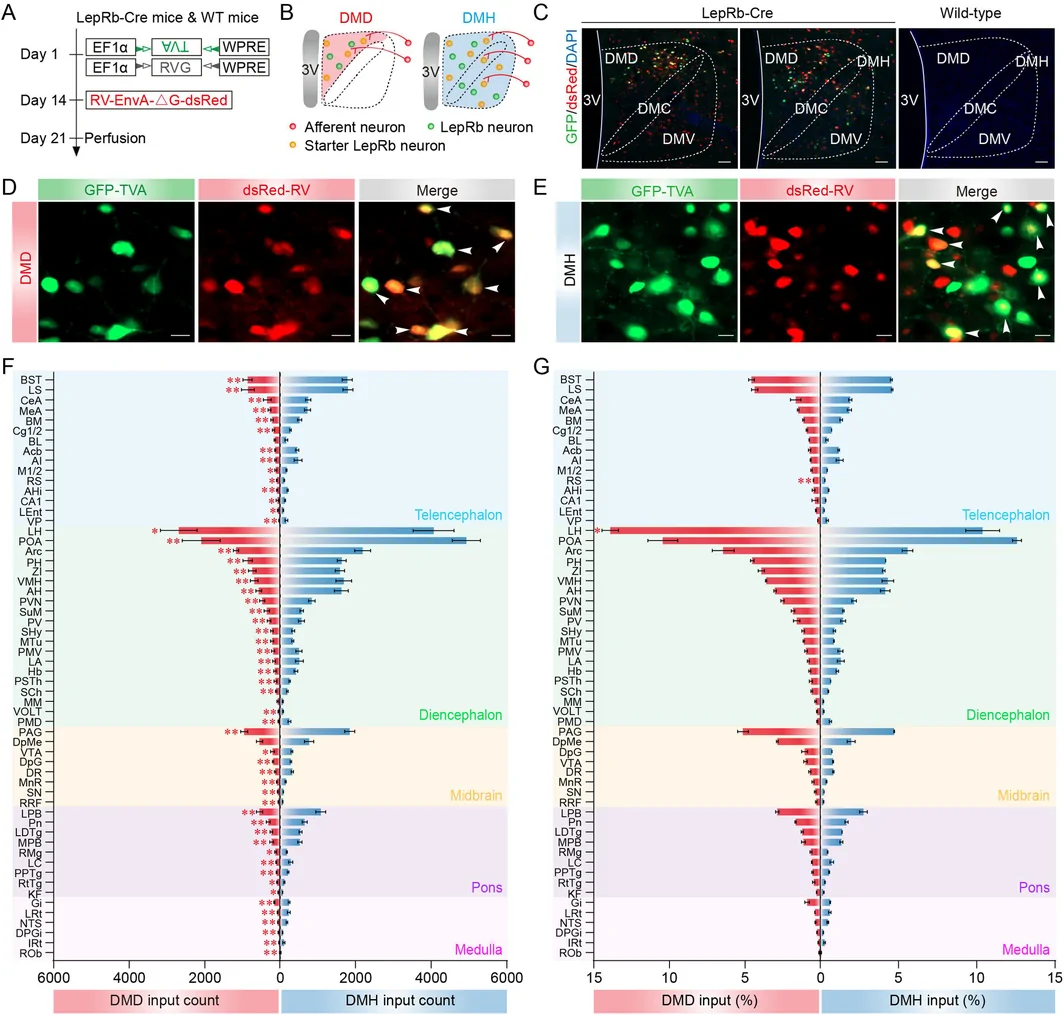
Monosynaptic inputs to DMD^LepRb^ and DMH^LepRb^ neurons. (A) Schematic of AAV helper viruses and RV injection timeline. (B) Coronal schematic of DMD and DMH viral infection: Red for RV labeling, green for AAV helper virus infection, and yellow for starter neurons. (C) Representative images of the DMD (left) and DMH (middle) show GFP‐ and dsRed‐expressing neurons in LepRb‐Cre mice, but not in wild‐type mice (right), following helper and RV injection. Scale bars, 50 μm. (D, E) Magnified views of DMD and DMH showing GFP‐expressing LepRb neurons (green), input neurons (red), and starter neurons (yellow, white arrowheads) in LepRb‐Cre mice. Scale bars, 10 μm. (F) Whole‐brain input counts of DMD^LepRb^ and DMH^LepRb^ neurons (*n* = 4; multiple unpaired *t*‐tests followed by Benjamini–Krieger–Yekutieli FDR correction for multiple comparisons). (G) Whole‐brain input proportions to DMD^LepRb^ and DMH^LepRb^ neurons (*n* = 4; multiple unpaired *t*‐tests followed by Benjamini–Krieger–Yekutieli FDR correction for multiple comparisons). Brain region abbreviations are detailed in Table 1. Data represent mean ± SEM. *q < 0.05, **q < 0.01.

### 3.3 Whole‐Brain Input Connectivity Features of DMD^LepRb^ and DMH^LepRb^ Neurons

To characterize whole‐brain input patterns of DMD^LepRb^ and DMH^LepRb^ neurons, we quantified dsRed‐labeled inputs on serial sections. Absolute numbers (Figure 2F) and proportion (Figure 2G) showed that both populations received bilateral monosynaptic inputs from 58 nuclei spanning the telencephalon, diencephalon, midbrain, pons, and medulla, with largely shared major sources. Dense input (> 5%) to DMD^LepRb^ neurons came from the lateral hypothalamic area (LH, 13.91%), POA (10.43%), Arc (6.43%), and periaqueductal gray (PAG, 5.13%); for DMH^LepRb^ neurons, from POA (12.57%), LH (10.37%), and Arc (5.58%). Moderate inputs (2%–5%) to DMD^LepRb^ neurons arose from the bed nucleus of the stria terminalis (BST, 4.56%), posterior hypothalamic area (PH, 4.52%), lateral septal nucleus (LS, 4.36%), zona incerta (ZI, 3.91%), VMH (3.59%), anterior hypothalamic area (AH, 3.01%), deep mesencephalic nucleus (DpMe, 2.86%), lateral parabrachial nucleus (LPB, 2.85%), and paraventricular hypothalamic nucleus (PVN, 2.51%); and to DMH^LepRb^ neurons from PAG (4.72%), LS (4.60%), BST (4.55%), VMH (4.32%), AH (4.15%), PH (4.18%), ZI (4.06%), LPB (2.76%), and PVN (2.16%). Sparse inputs (< 2%) came from 45 (DMD) and 46 (DMH) other regions (Figure 2G).

Despite shared input framework, further analysis revealed notable biases in the weights of specific connections. Although DMD^LepRb^ and DMH^LepRb^ neurons share largely overlapping input sources, we examined whether DMD differentially weights these shared afferents. We thus calculated a preference ratio for each input nucleus (DMD versus DMH). A ratio > 1 indicates that a given upstream nucleus provides a higher proportion of inputs to DMD^LepRb^ neurons relative to the overall DMH^LepRb^ population, suggesting that this pathway is more likely dominated by DMD. Conversely, a ratio < 1 indicates that the upstream nucleus provides a lower proportion of inputs to DMD relative to the overall DMH value, implying that these nuclei more likely project predominantly to the remaining DMH subdivisions. We calculated the input ratio between DMD^LepRb^ and DMH^LepRb^ neurons and categorized these regions into three types: DMD‐biased, balanced, and DMH‐biased. This approach identified input nodes specialized for DMD within the shared global afferent network (Figure 3A). A connectivity map shows representative DMD‐biased and DMH‐biased regions (Figure 3B). The results showed that DMD^LepRb^ neurons received stronger inputs from the basolateral amygdala (BL), retrosplenial cortex (RS), medial mammillary nucleus (MM), LH, DpMe, and Kölliker‐Fuse nucleus (KF); conversely, DMH^LepRb^ neurons received stronger inputs from the ventral pallidum (VP), dorsal premammillary nucleus (PMD), agranular insular cortex (AI), lateral reticular nucleus (LRt), lateroanterior hypothalamic nucleus (LA), and intermediate reticular nucleus (IRt). DMD‐biased inputs mainly involved emotional/arousal/defense regions, whereas DMH‐biased inputs enriched in visceral sensation, reward, and innate behaviors (Figure 3C,D). Together, DMD^LepRb^ and DMH^LepRb^ neurons share a conserved global input architecture, while differential connection weights of specific upstream nuclei form the structural basis for subregional connectional specialization.

**FIGURE 3:**
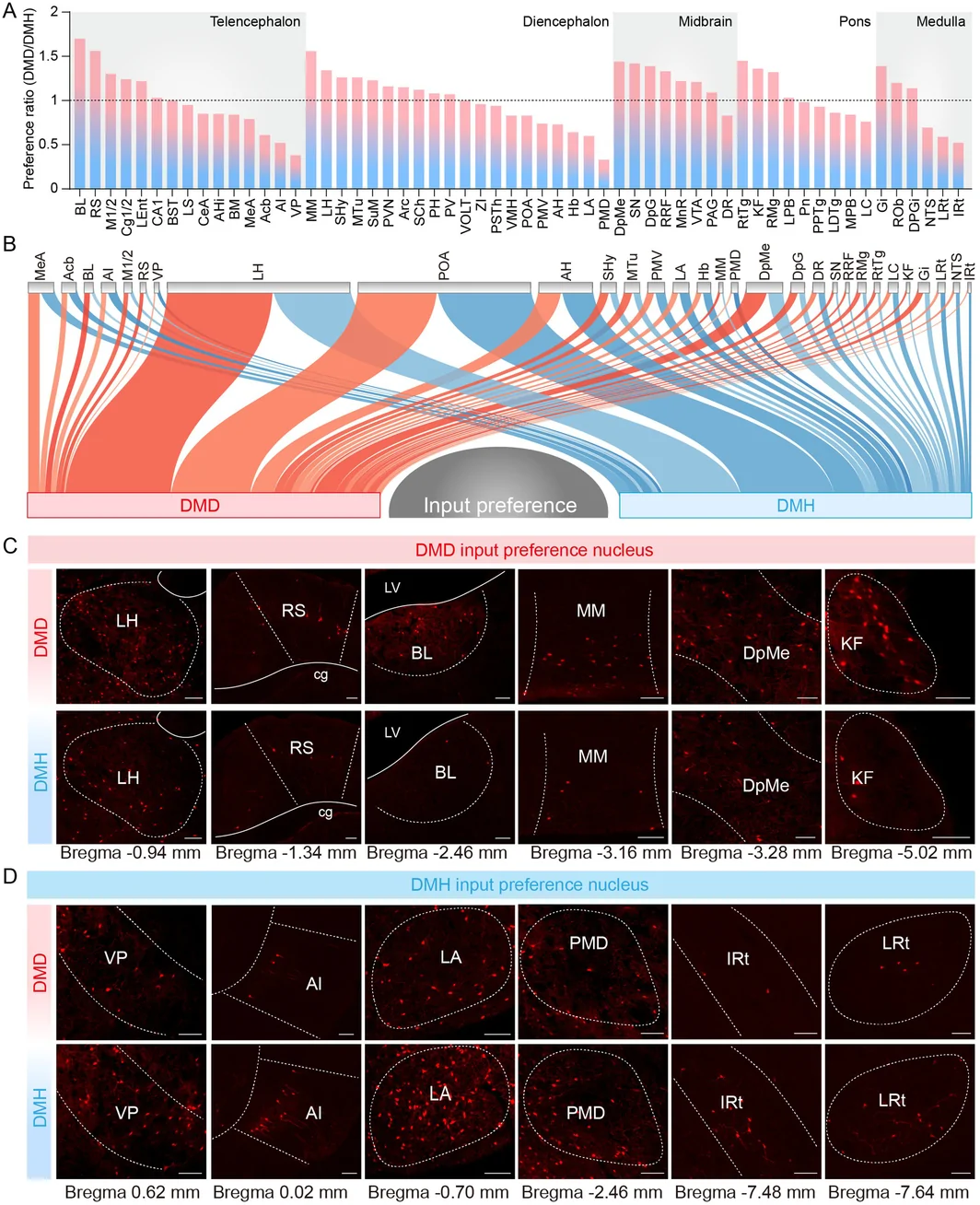
Preference analysis of afferent nuclei to DMD^LepRb^ and DMH^LepRb^ neurons. (A) Preference ratio of afferent inputs to DMD over DMH. The preference ratio was calculated as the proportion of each afferent input to DMD divided by that to DMH. Values > 1 indicate a bias toward DMD, whereas values < 1 indicate a bias toward DMH. These ratios are intended only to describe relative connectivity patterns and are not used to assign statistically significant bias categories. (B) Sankey diagram showing biased afferent inputs to DMD versus DMH. The width of each curve reflects the preference bias, while the color intensity corresponds to the input proportion. (C) Immunofluorescence of DMD^LepRb^‐biased afferent nuclei. Scale bars, 100 μm. (D) Immuno‐fluorescence of DMH^LepRb^‐biased afferent nuclei. Scale bars, 100 μm. Brain region abbreviations are detailed in Table 1.

### 3.4 Axonal Projection Patterns of DMD^LepRb^ and DMH^LepRb^ Neurons

To map downstream projections, AAV‐EF1α‐DIO‐EYFP was unilaterally injected into DMD or DMH (Figure 4A,C). Four weeks after injection, strong EYFP signals were detected in the somata (Figure 4B,D). Quantification focused on axonal boutons, excluding non‐bouton segments. We then quantified absolute bouton numbers (Figure 4E) and proportions (Figure 4F) per region. Both populations sent bilateral projections to 43 brain regions across the telencephalon, diencephalon, midbrain, pons, and medulla, with axons predominantly ipsilateral. Dense projections (> 5%) from DMD^LepRb^ neurons targeted Arc (27.28%), POA (13.62%), LH (8.05%), and PAG (5.33%). For DMH^LepRb^ neurons, dense targets included Arc (26.01%), POA (11.49%), LH (10.51%), PAG (9.68%), PVN (5.05%), and VMH (5.01%). Moderate projections (2%–5%) from DMD^LepRb^ neurons were observed in eight regions: PVN, AH, VMH, LA, paraventricular thalamic nucleus (PV), PH, periventricular hypothalamic nucleus (Pe), and BST. DMH^LepRb^ neurons targeted seven regions: premammillary nucleus, ventral part (PMV), PH, Pe, AH, PV, suprachiasmatic nucleus (SCh), and ventral tegmental area (VTA). Sparse projections (< 2%) were found in 31 and 30 regions, respectively (Figure 4F). These findings indicate that DMD^LepRb^ and DMH^LepRb^ neurons share major downstream targets within a conserved output framework.

**FIGURE 4:**
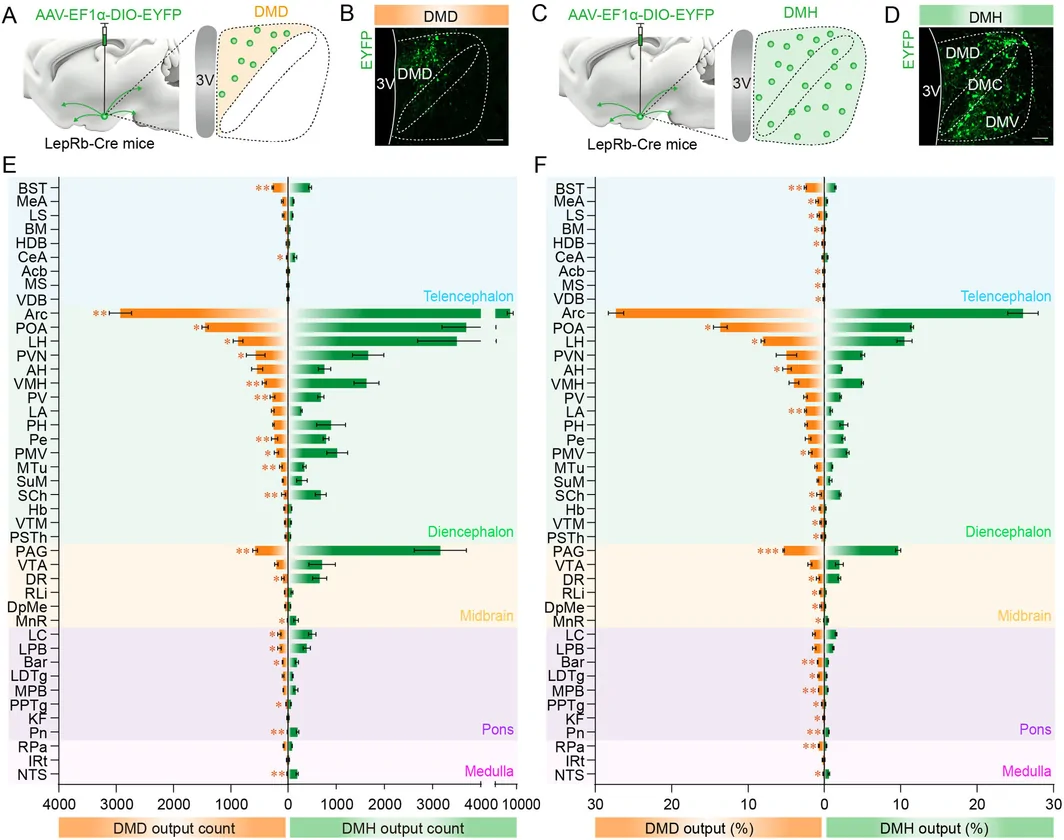
Efferent projections of DMD^LepRb^ and DMH^LepRb^ neurons. (A, C) Schematic diagram of anterograde tracing virus strategy. (B, D) Representative images of EYFP expression in the DMD and DMH. Scale bars, 100 μm. (E) Whole‐brain output counts of DMD^LepRb^ and DMH^LepRb^ neurons (*n* = 4; multiple unpaired *t*‐tests followed by Benjamini–Krieger–Yekutieli FDR correction for multiple comparisons). (F) Whole‐brain output proportions from DMD^LepRb^ and DMH^LepRb^ neurons (*n* = 4; multiple unpaired *t*‐tests followed by Benjamini–Krieger–Yekutieli FDR correction for multiple comparisons). Brain region abbreviations are detailed in Table 1. Data represent mean ± SEM. *q < 0.05, **q < 0.01, ***q < 0.001.

To further quantify projection preference, we applied the same ratio to identify DMD‐specialized output targets (Figure 5A,B). Despite sharing similar overall targets, this analysis revealed that DMD preferentially projected to the basomedial amygdaloid nucleus (BM), horizontal limb of the diagonal band (HDB), LA, DpMe, raphe pallidus nucleus (RPa), and IRt—regions involved in sleep–wake regulation, thermoregulation, and autonomic control. In contrast, DMH preferentially projected to the SCh, PAG, pontine reticular nucleus (Pn), dorsal raphe nucleus (DR), median raphe nucleus (MnR), and nucleus of the solitary tract (NTS)—regions associated with circadian rhythms, instinctive behaviors, and visceral sensory integration. Representative biased regions are shown in Figure 5C (DMD) and Figure 5D (DMH). These findings indicate that DMD^LepRb^ and DMH^LepRb^ neurons share a conserved global output framework, and subregional projection biases are implemented through differential weighting of shared downstream targets.

**FIGURE 5:**
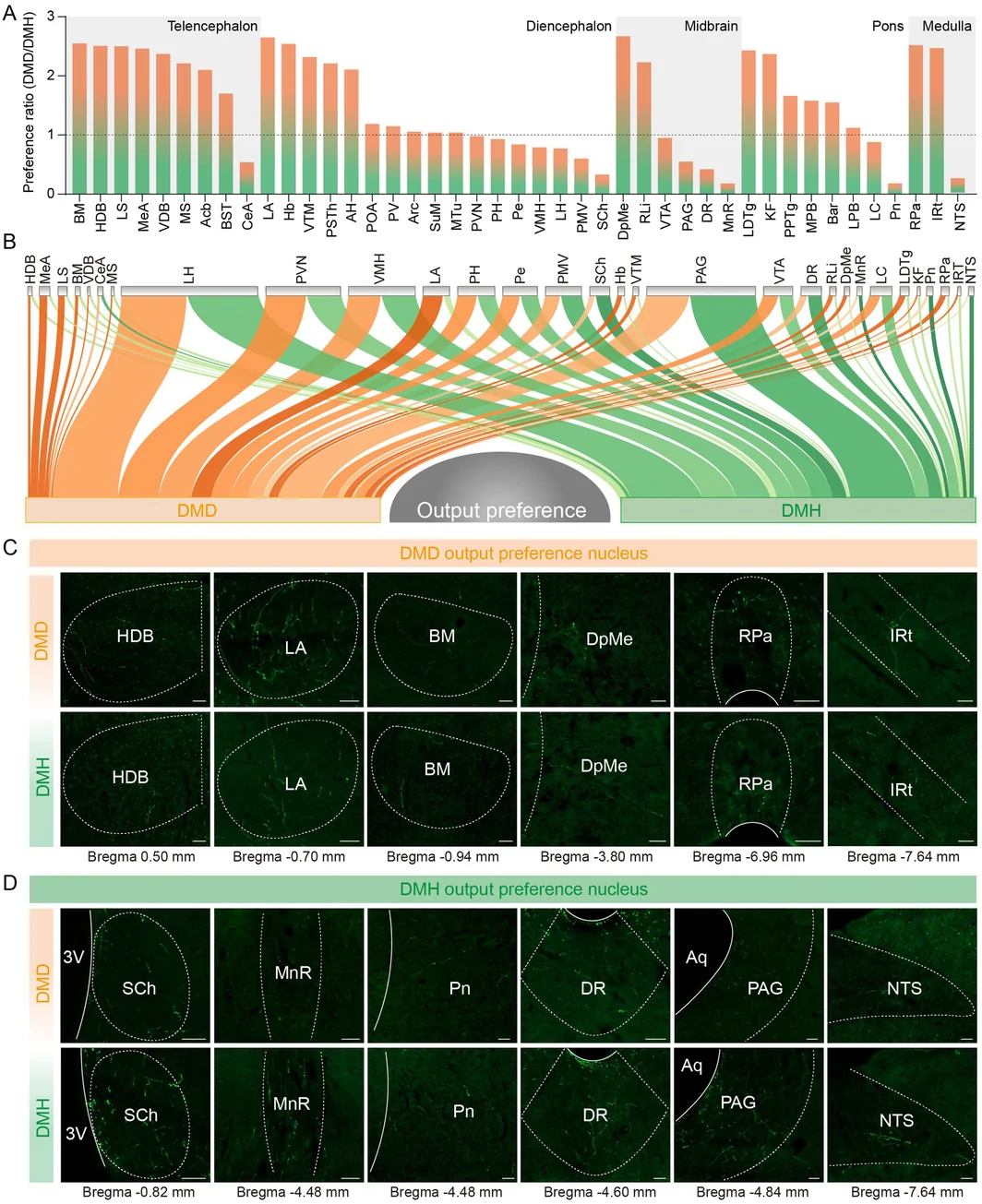
Preference analysis of efferent projections from DMD^LepRb^ and DMH^LepRb^ neurons. (A) Preference ratio of outputs from DMD over DMH. The preference ratio was calculated as the proportion of outputs from DMD divided by those from DMH. Values > 1 indicate a bias toward DMD output, whereas values < 1 indicate a bias toward DMH output. These ratios are intended only to describe relative connectivity patterns and are not used to assign statistically significant bias categories. (B) Sankey diagram showing biased efferent projections from DMD versus DMH. The width of each curve reflects the projection bias, while the color intensity corresponds to the output proportion. (C) Immunofluorescence of DMD^LepRb^‐biased efferent nuclei. Scale bars, 50 μm. (D) Immunofluorescence of DMH^LepRb^‐biased efferent nuclei. Scale bars, 50 μm. Brain region abbreviations are detailed in Table 1.

### 3.5 Comparison of Input–Output Distribution of DMD^LepRb^ and DMH^LepRb^ Neurons

To determine whether connectivity differences reflect distinct networks, we integrated whole‐brain input and output data for DMD^LepRb^ and DMH^LepRb^ neurons (Figure 6A,B). Both populations formed predominant bidirectional connections with the diencephalon, acting as hypothalamic hubs. However, regional weighting differed between the two populations in their outputs: DMD^LepRb^ neurons preferentially targeted the telencephalon, whereas DMH^LepRb^ neurons preferentially targeted the midbrain (Figure 6B). In contrast, no significant differences were observed in their input patterns across major brain divisions (Figure 6A). Correlation analyses of major input–output nuclei (Figure 6C) showed strong positive correlations (*r* ≥ 0.95) between DMD^LepRb^ and DMH^LepRb^, with highly consistent key data. Thus, both populations share a highly homologous global connectivity architecture, operating on a shared core framework rather than as independent parallel circuits.

**FIGURE 6:**
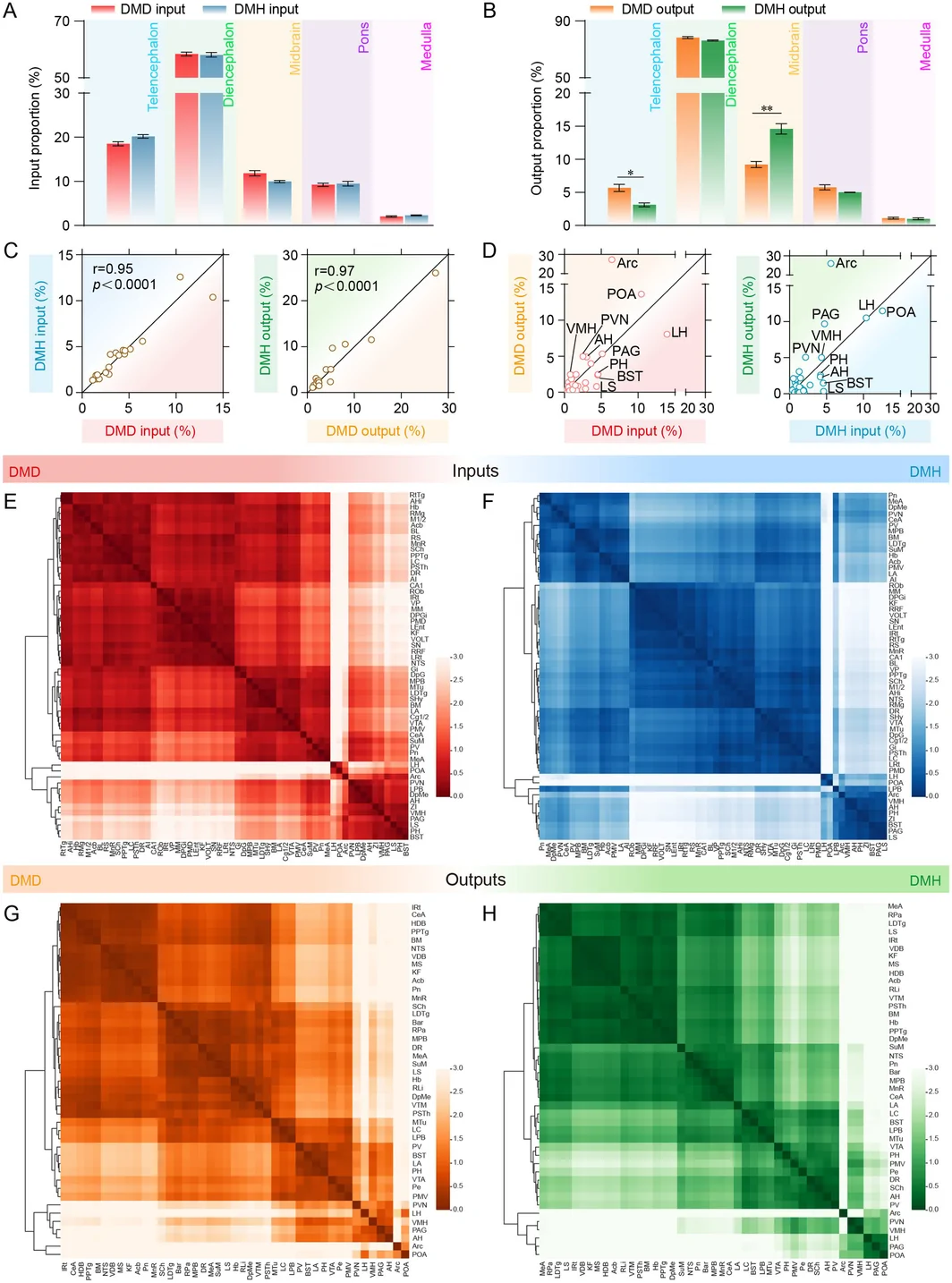
Comparative analysis of whole‐brain input and output of DMD^LepRb^ and DMH^LepRb^ neurons. (A) Comparison of input distributions to DMD^LepRb^ and DMH^LepRb^ neurons across five major brain subdivisions (*n* = 4, multiple unpaired *t*‐tests followed by Benjamini–Krieger–Yekutieli FDR correction for multiple comparisons). (B) Comparison of output distributions from DMD^LepRb^ and DMH^LepRb^ neurons across five major brain subdivisions (*n* = 4, multiple unpaired *t*‐tests followed by Benjamini–Krieger–Yekutieli FDR correction for multiple comparisons). (C) Comparison of inputs (left) and outputs (right) between the DMD^LepRb^ and DMH^LepRb^ neurons. Nuclei with a ratio greater than 1 were selected for analysis. (D) Input and output proportions of nuclei that are bidirectionally connected with both DMD^LepRb^ (left) and DMH^LepRb^ (right) neurons. (E, F) Hierarchical clustering analysis (Euclidean distance) of all inputs to DMD^LepRb^ and DMH^LepRb^ neurons. (G, H) Hierarchical clustering analysis (Euclidean distance) of all output nuclei from DMD^LepRb^ and DMH^LepRb^ neurons. The color scales in E–H indicate Euclidean distance, where darker colors represent smaller distances (higher similarity) and lighter colors represent larger distances (lower similarity). Brain region abbreviations are detailed in Table 1. Data represent mean ± SEM. *q < 0.05, **q < 0.01.

Given this global homology, we examined specific‐nucleus connection strengths via input–output bias scatter plots (Figure 6D). Some regions showed similar input–output ratios across groups: Arc consistently above the diagonal (strongest common output target), while BST, PH, and LS remained below (predominant input‐receiving). Others displayed distinct distributions: LH showed input bias in DMD^LepRb^, balanced in DMH^LepRb^; POA and AH exhibited the opposite pattern (output bias in DMD^LepRb^ versus input bias in DMH^LepRb^); and PAG was balanced in DMD^LepRb^ but output‐biased in DMH^LepRb^. These varied directions reveal input–output polarization across the two populations, indicating that different nuclei exhibit distinct connection biases.

To further assess spatial heterogeneity, we performed hierarchical clustering on input (Figure 6E,F) and output nuclei (Figure 6G,H) of DMD^LepRb^ and DMH^LepRb^ neurons. On the input side, PVN, LPB, DpMe, and AH formed tight, dark clusters in DMD^LepRb^ but dispersed into distinct, lighter branches in DMH^LepRb^, indicating reduced input homogeneity in DMH^LepRb^; however, LH and POA remained in consistent clusters. On the output side, DR, medial amygdaloid nucleus (MeA), supramammillary nucleus (SuM), and LS exhibited high similarity in DMD^LepRb^ neurons but scattered in DMH^LepRb^ neurons, whereas LH and PAG shared common clusters. These results confirm that DMD^LepRb^ neurons possess a more compact and coherent intrinsic network organization than DMH^LepRb^ neurons. Collectively, these data demonstrate that DMD^LepRb^ neurons belong to the unified DMH network system rather than forming an isolated independent circuit, and that local connection‐weight differences underlie the connectional divergence between subdivisions.

## 4 Discussion

### 4.1 Comparison With Previous Tracing Studies

Classical anatomical studies using conventional tracers, including cholera toxin subunit B (CTb) [28], biotinylated dextran amine (BDA) [29], Fluoro‐Gold (FG), and *Phaseolus Vulgaris* leucoagglutinin (PHA‐L) [30, 31], revealed DMH connectivity with hypothalamic, midbrain, and brainstem nuclei, but lacked cell‐type specificity and were susceptible to passing fibers and polysynaptic contamination. Cre‐loxP viral tracing enabled cell‐type‐specific analyses. Zhang et al. demonstrated that DMH^LepRb^ neurons receive POA inputs and project to POA, BST, Arc, PAG, and RPa [32], however, a cell‐type‐specific connectomic comparison across DMH subregions remains lacking. Here, we combined monosynaptic rabies retrograde tracing, Cre‐dependent anterograde tracing, and whole‐brain quantitative analysis to compare the input–output organization of DMD^LepRb^ neurons with that of the DMH^LepRb^ population. This approach provides cell‐type selectivity, monosynaptic specificity, and a brain‐wide assessment. Rather than defining DMD and DMH as separate systems, our findings reveal that connectional diversification within the DMH emerges through locally biased connectivity embedded within a conserved homeostatic network architecture.

### 4.2 DMH^LepRb^ Neurons Form a Broad Brain‐Wide Homeostatic Integration Network

Our results demonstrate that DMH^LepRb^ neurons establish extensive connections across the telencephalon, diencephalon, midbrain, pons, and medulla, with the diencephalon as the major bidirectional hub, aligning with classical DMH connectivity studies [30, 31]. At the input level, DMH^LepRb^ neurons receive prominent monosynaptic inputs from POA, LH, Arc, PAG, BST, and VMH—regions involved in thermoregulation, energy balance, feeding, arousal, defensive behaviors, and autonomic control [33, 34, 35, 36, 37, 38]. At the output level, DMH^LepRb^ neurons project extensively to Arc, POA, LH, PAG, and PVN, with the PVN serving as a major relay for hypothalamic–pituitary–adrenal axis activation and autonomic output [39]. This extensive connectivity with both the limbic system and brainstem provides a structural substrate for translating emotional states into visceral responses. Previous studies show that DMH^LepRb^ neurons regulate feeding via GABAergic projections to the Arc [24]; coordinate metabolism through distinct subpopulations projecting to the Arc and RPa [15]; and modulate sleep‐related respiration, REM sleep [18], and circadian feeding rhythms [13]. Thus, DMH^LepRb^ neurons constitute a multimodal integration network linking metabolic status with autonomic, respiratory, sleep, and behavioral states.

### 4.3 Conserved Global Architecture With Local Specialization: An Organizational Principle of the DMH

As the DMD is an anatomical subregion of the DMH, overlapping upstream and downstream nuclei are expected, confirming that DMD circuits are embedded in the shared DMH‐wide connective framework rather than forming independent parallel pathways. Despite this conserved global architecture, quantitative analyses revealed marked connection‐weight biases across whole‐brain input–output networks. DMD^LepRb^ neurons preferentially connect with nuclei regulating arousal, defense, and sympathetic thermogenesis, whereas DMH^LepRb^ neurons show stronger weighting in metabolic, neuroendocrine, and circadian regions. Within the DMH, which comprises the DMD, dorsomedial hypothalamus compact part (DMC), and DMV subdivisions, this divergence indicates that stress defense and sympathetic thermogenesis are preferentially mediated by the DMD, while feeding and neuroendocrine control are likely dominated by the DMC and DMV subdivisions.

This pattern of a shared network backbone with subregion‐specific weight biases mirrors organizational principles described in the VMH [26], PAG [40], and limbic circuits [27] where functional diversity arises from redistributed information flow within a common anatomical framework. Extending this principle to the DMH, our data demonstrate that hypothalamic subnuclear specialization does not require fully segregated circuits: conserved architecture ensures coordinated homeostatic regulation, while differential weighting enables subregions to govern distinct physiological processes. We therefore propose a hierarchical DMH organization: a shared connectivity framework supports global integrative function, and local connection‐weight biases drive subregional specialization. Our data validate this principle using the functionally well‐defined DMD as a test case.

### 4.4 Input–Output Connectivity Biases and Complementary Functional Organization of DMD and Remaining DMH Subdivisions

As the whole‐DMH dataset covers all three subdivisions (DMD, DMC, and DMV), weight differences between DMD^LepRb^ and DMH^LepRb^ neurons indirectly reflect connectivity preferences of the remaining subdivisions (predominantly DMC and DMV). At the input level, the DMD receives enriched afferents from emotion/arousal/defense regions (BL, RS, MM, LH, DpMe, KF) [41, 42, 43, 44], while remaining subdivisions show stronger inputs linked to instinctive behavior, reward, and visceral sensation (VP, PMD, IRt, AI, LA, LRt) [45, 46, 47]. Efferent projections follow a parallel complementary pattern: the DMD preferentially targets sleep–wake, thermoregulatory, and autonomic nuclei (BM, HDB, LA, DpMe, RPa, IRt) [48, 49, 50, 51], whereas remaining subdivisions weight more heavily toward circadian regulation, neuroendocrine control, and visceral sensory integration regions (SCh, PAG, Pn, DR, MnR, NTS) [52, 53, 54].

Functionally, this profile defines the DMD as a specialized gateway for higher‐order stress signals, integrating arousal/defense inputs to autonomic effectors without disrupting DMH network coherence. This arrangement offers a network‐topological mechanism for hypothalamic subnuclear diversity: functional partitioning arises from differential weighting of shared circuits, independent of full anatomical segregation. Notably, these connectivity features of non‐DMD subdivisions are inferred from group comparisons rather than direct anatomical tracing. Future studies with subregion‐specific labeling of DMV and DMC neurons are required for validation.

### 4.5 Limitations and Future Directions

Several limitations should be acknowledged. First, rabies tracing reveals anatomical connectivity, yet does not confirm functional transmission or physiological significance. Moreover, we did not directly test the behavioral, autonomic, or metabolic roles of DMD^LepRb^ neurons. Future studies combining projection‐specific manipulations (e.g., optogenetics, electrophysiology) with behavioral and physiological assays are needed to establish the precise functional relevance of these circuits. Second, while we quantified axonal varicosities as candidate presynaptic sites, varicosities do not always equate to functional synapses, necessitating future optogenetic‐electrophysiological and presynaptic marker validation. Third, although both sexes were included, systematic quantitative comparisons between sexes were not performed; whether these circuits exhibit sex‐dependent differences requires future investigation. Fourth, the DMH contains three subnuclei, but our comparison was between DMD^LepRb^ and whole‐DMH^LepRb^ populations. Thus, connectivity features of DMV^LepRb^ and DMC^LepRb^ neurons may be diluted in the whole‐DMH dataset. Subnucleus‐specific tracing is needed to fully resolve DMH heterogeneity. Finally, unilateral viral injections primarily reveal ipsilateral projections; given that many DMH‐regulated functions involve bilateral coordination, contralateral pathways may be underestimated and warrant bilateral tracing.

## 5 Conclusions

This study systematically maps whole‐brain input–output organization of DMD^LepRb^ and DMH^LepRb^ neurons, showing that the DMH achieves functional diversification through local connectivity biases within a conserved global architecture. Our findings provide an anatomical framework for DMH functional heterogeneity and circuit mechanisms coordinating metabolic, autonomic, and behavioral states.

## Author Contributions

Study design and manuscript drafting/revising: Fang Yuan and Mengchu Zhu. Running of the experiments, acquisition of the data, and figure drawing: Yaxin Hao, Xiang Zhang, and Yongqiang Chen. Acquisition of the data and statistical analysis: Danyang Zhao, Haixing Zhong, Heming Li, and Minghui Ren. All authors have read and approved the final version of the manuscript.

## Funding

This work was supported by the National Natural Science Foundation of China, 32671478 and 82170104. Hebei Province Yanzhao Golden Platform Project, HJZD202510. Natural Science Foundation of Hebei Province, H2025206880. Graduate Innovation Funding Program of Hebei Province, CXZZBS2024114.

## Ethics Statement

All experimental procedures were approved by the Animal Care and Ethics Committee of Hebei Medical University (Approval No. Hebmu‐P2023052) and were conducted in accordance with the Guide for the Care and Use of Laboratory Animals.

## Conflicts of Interest

The authors declare no conflicts of interest.

## Supporting information


**Figure S1:** Quality control and cell clustering characterization of single‐cell transcriptomic profiling in the DMH nucleus. (A) Violin plots illustrating the distribution of three quality control parameters for individual cells isolated from the DMH: number of detected genes per cell (left), UMI counts per cell (middle), and mitochondrial gene transcript percentage (right). (B) UMAP‐based dimensionality reduction plot of single cells. Each dot represents an individual cell, and distinct colors denote different cell subclusters. (C) Feature expression plots of representative marker genes projected onto the UMAP space. From left to right: *Slc17a6* (excitatory neuronal marker), *Slc32a1* (inhibitory neuronal marker), and *Lepr*. Color intensity from light gray to red represents gene expression levels from low to high (red, high expression).

## Data Availability

The data that support the findings of this study are available from the corresponding author upon reasonable request.
